# Oxidative stress and metabolic biomarkers in patients with Psoriasis

**DOI:** 10.5937/jomb0-45076

**Published:** 2024-01-25

**Authors:** Mirjana Bakić, Aleksandra Klisić, Gordana Kocić, Hristina Kocić, Vesna Karanikolić

**Affiliations:** 1 Clinical Center of Montenegro, Clinic for Dermatovenerology, Podgorica, Montenegro; 2 University of Montenegro, Faculty of Medicine, Podgorica, Montenegro; 3 Primary Health Care Center, Center for Laboratory Diagnostics, Podgorica, Montenegro; 4 University of Nis, School of Medicine, Department of medical Biochemistry, Nis; 5 University of Nis, School of Medicine, Clinic for Skin Diseases of the Clinical Center Nis, Nis

**Keywords:** cytokines, inflammation, oxidative stress, psoriasis, citokini, inflamacija, oksidativni stres, psorijaza

## Abstract

**Background:**

Psoriasis is an autoinflammatory disease that affects not only skin but multiple organs thus being associated with many comorbidities. Oxidative stress and inflammation play the major role in the pathogenesis of this disease. Studies that examined by-products of oxidative stress in psoriasis show discrepant results. Hence, we aimed to examine the oxidative stress, inflammation and metabolic markers and to explore their potential relationship with disease severity in patients with psoriasis.

**Methods:**

This case-control study comprised of 35 patients with psoriasis and 35 age, sex and body mass index-matched healthy controls. Metabolic and oxidative stress biomarkers [i.e., malondialdehyde (MDA), advanced oxidation protein products (AOPP), and catalase (CAT)] were measured. The principal component analysis (PCA) was employed to reduce the number of measured variables into smaller number of factors. PCA factors were subsequently used in logistic regression analysis for severe psoriasis prediction.

## Abstract (sr)

**Uvod:**

Psorijaza je autoinflamatorno oboljenje koje zahvata ne samo kožu, već i brojne organe i povezano je sa brojnim komorbiditetima. Oksidativni stres i inflamacija igraju glavnu ulogu u patogenezi ovog oboljenja. Kada su u pitanju biomarkeri oksidativnog stresa, studije pokazuju oprečne rezultate. Stoga je cilj ovog istraživanja bio da se ispita potencijalna povezanost biomarkera oksidativnog stresa, inflamacije i metaboličkih parametara sa težinom bolesti kod pacijenata sa psorijazom.

**Metode:**

Ova studija slučajeva i kontrola obuhvatila je 35 pacijenata sa psorijazom, koliko i zdravih ispitanika uparenih po godinama starosti, polu i indeksu telesne mase. Metabolički i biomarkeri oksidativnog stresa [tj., malon dialdehid (MDA), produkti uznapredoovale oksidacije proteina (AOPP) i katalaza (CAT)] su mereni. Analiza glavnih komponenti (PCA) je primenjena da smanji broj varijabli u manje grupe faktora koji su dalje korišćeni u logističkoj regresionoj analizi za predikciju težine bolesti.

**Rezultati:**

Veće vrednosti AOPP (p<0,01) i aktivnosti CAT (p<0,001), bez postojanja razlike u MDA vrednostima uočene su kod pacijenata sa psorijazom u pore|enju sa kontrolnom grupom. Multivarijantna binarna logistička regresiona analiza pokazala je da je kombinacija metaboličkih faktora (tj., glukoze i triglicerida) i renalnih faktora (tj., kreatinin i urea) najbolji model koji predvi|a Psoriasis Area and Severity Indeksa (PASI) >10, dok je oksidativni stres-hepatični faktor (tj., MDA i alanin aminotransferaza) najbolji prediktor Dermatology Life Quality Indeksa (DLQI) >20.

**Zaključak:**

"Multimarker" pristup (metabolički, renalni i oksidativni stres-hepatični faktor) se pokazao boljim prediktorom težine bolesti kod pacijenata obolelih od psorijaze nego bilo koji pojedinačni ispitivani biomarker.

## Introduction

Psoriasis is an autoinflammatory disease that affects not only skin but multiple organs thus being associated with many comorbidities [1]. The prevalence of psoriasis differs between the regions, i.e. between 0.51% and 11.43% in the adult population which makes this disease as significant global health concern [1] [2].

Although incompletely enlightened, it is assumed that poor regulation of T cells that are triggered by cells of innate immunity (i.e., macrophages, neutrophils, keratinocytes, dendritic cells) or by an unknown autoantigen are the initiators of this process [3]. Like in many other chronic diseases (i.e., diabetes mellitus, hypertension, obesity, cardiovascular disease, etc.) [4] [5] [6], oxidative stress and inflammation play the major role in the pathogenesis of psoriasis [7] [8] [9]. The impaired redox balance in favor of pro-oxidants [i.e., reactive oxygen species (ROS) and reactive nitrogen species (RNS)] leads to oxidative modifications and destruction of lipids, proteins and DNA structures in almost all cells and tissues [8].

The increased secretion of proinflammatory cytokines [e.g., tumor necrosis factor alpha (TNF-α), interleukins (IL)-1β, IL-6, IL-17, IL-22 and IL-12/23)] favors the hyperproliferation and abnormal differentiation of epidermis [1] [3]. The enhanced inflammatory response further promotes ROS/RNS production and diminishes already reduced antioxidant defence system, thus making a vicious circle between oxidative distress and inflammatory processes in the pathophysiological cascade of psoriasis [1] [3].

The proinflammatory cytokines mentioned above further influence lipid metabolism [1]. Cholesterol metabolites affect the keratinocytes function, but also the inflammation and immune response, thus further contributing to pathogenesis of this disease [1].

Given the fact that psoriasis is a systemic disease, biomolecules’ alterations related to cholesterol metabolism are correlated with comorbidities such as obesity, dyslipidemia, hypertension, insulin resistance, non-alcoholic fatty liver disease (NAFLD) and diabetes [1] [9] [10]. Patients with psoriasis are also at increased risk of chronic kidney disease (CKD) and end-stage renal disease since proinflammatory cytokines influence renal hemodynamics, favor the retention of sodium and lead to hypertension onset and renal injury [11].

However, studies that examined by-products of oxidative stress in psoriasis show discrepant results [12] [13] [14] [15] [16] [17] [18] [19] [20]. Hence, the clarification of complex pathophysiological processes underlying psoriasis is of urgent need. The aim of this study was to examine the oxidative stress, inflammation and metabolic markers severity in patients with psoriasis as compared with healthy controls patients and explore their potential relationship with disease severity in patients with psoriasis.

## Materials and methods

### Patients

This case-control study comprised of 35 patients with psoriasis and 35 age, sex and body mass index (BMI)-matched healthy controls. The Institutional Ethics Committee approved the study protocol. The study was conducted following the ethical principles of Helsinki Medical Declaration and each participant signed an informed consent.

The examinees have filled in the questionnaires (i.e. consisted of demographic data, medication useand lifestyle habits), whereas the blood sampling procedure and the anthropometric measurements (i.e., body height, weight and BMI) were conducted the same morning.

The diagnosis of psoriasis was the inclusion criterion for cases. Healthy subjects were free of any disease and medication use which were the inclusion criteria for control group, whereas the voluntarily acceptance to participate in the study was the inclusion criterion for all the participants. Participants with malignancies, cardiovascular diseases, stroke, mental disorders, as well as those who used antioxidant supplements were excluded from the study.

The severity of skin disease was presented with the Psoriasis Area and Severity Index (PASI) [17] [21]. The evaluation of the influence of skin disease on Quality of life was determined with the Dermatology Life Quality Index (DLQI), i.e. self-reported questionnaire consisted of 10-items. The DLQI ranged from 0 (no impairment) to 30 (maximal impairment) [22].

### Methods

Blood sampling procedure was conducted in the morning after fasting state of at least 8 hours. The samples were obtained in serum separator and clot activator tubes. After being left to clot for about 30 minutes, the samples were then centrifuged for the biochemical analyses. Metabolic parameters, i.e. glucose, total cholesterol (TC), low-density lipoprotein cholesterol (LDL-c), triglycerides (TG), high-density lipoprotein cholesterol (HDL-c), urea, uric acid, creatinine, alanine aminotransferase (ALT), aspartate aminotransferase (AST) and C-reactive protein (CRP) were measured on Roche Cobas c501 chemistry analyzer (Roche Diagnostics GmbH, Mannheim, Germany).

Parameters of oxidative stress were measured spectrophotometrically. Advanced oxidation proteinproducts (AOPP) were measured following the recommendations of the Witko-Sarsat method, by a reaction with potassium iodide and glacial acetic acid [23]. Measurement of MDA was based on the determination of thiobarbituric acid reactive substances by thiobarbituric acid test [24].

Catalase (CAT) was measured by the release of oxygen from hydrogen peroxide (H_2_O_2_), based on the formation of the stable complex with ammonium molybdate [25].

### Statistical analysis

Statistical analysis was determined using SPSS statistical package (version 18.0 for Windows, SPSS,Chicago, IL, USA). Data are presented as mean±standard deviation (SD), median (interquartile range), or counts and percentages. Differences between groups were evaluated with Student’s t-test and Mann-Whitney U test for continuous data. Chi-square test was employed to test the differences between categorical data. A correlation analysis with Spearman’s (p) correlation coefficient was used to determine the relationships between examined variables. In order to reduce the number of measured variables into smaller number of factors we employed principal component analysis (PCA) with varimax rotation and extraction parameter eigenvalue >1 and factors coefficients’ smaller than 0.500 suppression. PCA produced scores were saved as variables for subsequent use in logistic regression analysis. P level <0.05 was set as the statistical significance.

## Results

Table 1 shows socio-demographic, clinical data and biochemical parameters in patients with psoriasis and gender, age and BMI-matched healthy subjects without psoriasis (i.e., control group) aiming to establish the general difference between the two opposed groups. This is important because of possibility to readily follow patients’ status change by some routinely measured parameters and also to find relationship between disease process and metabolic changes.

**Table 1:** Clinical and biochemical parameters in patients with psoriasis and healthy control group.

Parameters	Control group	Patients with psoriasis	P
Age (years)	56 (49–61)	48 (40–63)	0.397
Gender (male/female) n (%)	20/15 (57/43)	20/15 (57/43)	0.595
BMI (kg/m^2^)	27.2±3.8	27.1±4.4	0.879
Smokers, n (%)	10 (29)	16 (46)	0.011
Disease duration (years)	/	8 (5.0–15.5)	/
Therapy local/Methotrexate, n	/	30/5 (85.2/14.3)	
Skin change	/	21/21/7/5/4/2/2(60/60/20/14/11/6/6)	/
Comorbidities, n (%)			
Without	0	14 (40)	/
Hypertension	0	16 (46)	
Hypertension+hyperlipidemia	0	5 (14)	
PASI	/	15 (10–18)	/
DLQI	/	20 (18–25)	/
Glucose (mmol/L)	5.4 (5.1–5.9)	5.30 (4.9–5.9)	0.634
Creatinine (μmol/L)	70 (61-81)	65 (58–77)	0.452
Urea (mmol/L)	5.1 (4.3-5.6)	5.1 (4.0–6.0)	0.986
Uric acid (μmol/L)	281 (236-335)	310 (242–340)	0.474
TC (mmol/L)	5.29 (4.52–6.06)	5.26 (4.52–6.14)	0.729
LDL-c (mmol/L)	3.37 (2.64–3.90)	2.95 (2.19–3.70)	0.290
HDL-c (mmol/L)	1.41 (1.16–1.73)	1.36 (1.07–1.59)	0.381
TG (mmol/L)	1.44 (1.05–1.96)	1.64 (0.97–2.33)	0.518
TG/HDL-c ratio	0.95 (0.67–1.57)	1.12 (0.66–1.72)	0.414
non-HDL-c (mmol/L)	4.01 (3.14–4.50)	3.77 (2.98–4.76)	0.860
ALT (U/L)	21 (18–25)	24 (20–34)	0.006
AST (U/L)	23 (17–30)	27 (18–46)	0.184
CRP (mg/L)	1.45 (0.58–2.46)	1.80 (0.70–4.35)	0.167

Smokers’ percent was significantly higher in psoriasis patients compared to control group (P=0.011). A total of 60% of patients with psoriasis had comorbidities, i.e. hypertension and hyperlipidemia. Metabolic parameters were not different between patients and control group, except for ALT activity which was significantly higher in the patients group, but this enzyme’s activity values were still in the reference range and accordingly without clinical significance (Table 1).

The disease duration in a psoriasis group of patients varied from 6 months to 25 years (with median values of 8 months). The prevalent skin damage was localized on the trunk and upper and lower extremities (66% of patients), capillitium (in 20% of patients), nails (14%), elbows (11%), hands and face (about 6% the both). Local therapy was implemented in most of the patients, while in 14% of patients the antimetabolite drug methotrexate was the main therapy option. More than 70% of patients (n=25) had PASI score above 10, which is assumed as severe psoriasis cut-off value. Accordingly, more than 90% of patients had DLQI score higher than 10, which is also a sign of disease gravity.

**Figure 1:**
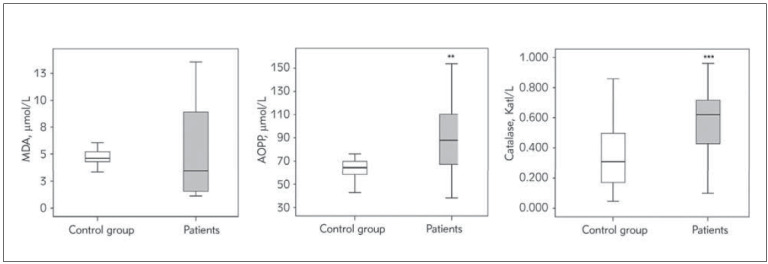
Concentration redox status parameters, MDA, AOPP and catalase in psoriasis patients and control group. **, *** P<0.01, 0.001 vs. control group

Redox status parameters analysed in this current study (i.e., MDA, AOPP and CAT) are presented in the Figure 1.

We noticed significantly higher AOPP concentration and antioxidant enzyme CAT activity in psoriasis patients compared to healthy subjects, while MDA did not differ between the two study groups.

More than a half of patients have comorbidities so we compared all measured parameters according to comorbidity status in patients with psoriasis. Results are showed at the Table 2 (i.e., only parameters which showed significant difference according to comorbidity status are presented).

**Table 2:** The influence of comorbidities on biochemical parameters in psoriasis patients. P-from the Mann-Whitney U test

Parameter	Without comorbidities<br>n=14	With comorbidities<br>n=21	P
Creatinine (μmol/L)	70 (63–84)	57 (55–70)	0.006
TC (mmol/L)	4.70 (4.12–5.63)	6.13 (5.75–6.88)	0.002
LDL-c (mmol/L)	2.56 (2.13–3.34)	3.77 (3.47–4.03)	0.003
TG (mmol/L)	1.23 (0.81–2.02)	2.12 (1.64–2.78)	0.031
non-HDL-c (mmol/L)	3.43 (2.66–3.90)	4.62 (4.28–5.26)	0.001

Significantly lower creatinine concentration in patients with comorbidities compared to patients without other diseases except psoriasis was observed. Other significant difference was noticed regarding lipid status parameters, with dyslipidemia pronounced in patients with comorbidities, which is not surprising because the dominant additional diseases were dyslipidemia and hypertension.

Correlation analysis between redox status parameters, metabolic parameters and psoriasis severity scores was performed by using Spearman’s nonparametric test (Table 3).

**Table 3:** Correlation between redox status parameters, metabolic parameters and psoriasis severity scores. Spearman’s coefficient correlation (Rho) with level of significance<br>*, **, *** P<0.05, 0.01, 0.001; ns-non-significant

Parameter	MDA (μmol/L)	AOPP (μmol/L)	CAT (Kat/L)	PASI score	DLQI score
AOPP (μmol/L)	ns		-0.382*	ns	ns
CAT (Kat/L)	ns	-0.382*		ns	ns
PASI score	ns	ns	ns		0.415*
DLQI score	ns	ns	ns	0.415*	
Age (years)	ns	ns	ns	ns	-0.448**
BMI (kg/m^2^)	0.348*	ns	ns	ns	ns
Urea (mmol/L)	ns	ns	ns	-0.423*	-0.389*
HDL-c (mmol/L)	ns	-0.356*	0.424*	ns	ns
TG (mmol/L)	ns	0.335*	ns	ns	ns
TG/HDL-c	ns	0.403*	ns	ns	ns
CRP (mg/L)	ns	ns	-0.538***	ns	ns

Significant negative correlation between some redox status parameters (i.e., AOPP and CAT) were observed. MDA showed significant positive correlation with BMI. AOPP significantly correlated with lipid parameters (i.e., negative with HDL-c, and positive with TG and TG/HDL-c ratio). Catalase showed significant positive correlation with HDL-c and negative with CRP.

We also observed significant positive correlation between PASI and DLQI scores, but also significant negative correlation between PASI and urea concentration. DLQI score was negatively correlated with age and urea concentration (Table 3).

Kaiser-Meyer-Olkin measure (0.503) confirmed good sample adequacy, so as Bartlett’s test of sphericity (P=0.002), as a main conditions for PCA analysis. The analysis extracted 4 principal components (factors) which explained 64% of total variability. Four main factors were as follows: Oxidative stress-inflammation related factor (HDL-c, CAT, AOPP and CRP explained 18% of total variability), renal function related factor (creatinine and urea explained 17% of total variability), metabolic related factor (glucose and TG with 16% of explained variability) and oxidative stress-hepatic related factor (MDA and ALT with 13% of the total variability). The results are presented at the Table 4.

**Table 4:** PCA extracted factors from metabolic and redox status parameters in a group of psoriasis patients.

Factor	Variables with Loadings	Factors’ percent of<br>total variability
Oxidative stress-inflammation<br>related factor	HDL-c -0.747<br>CAT -0.684<br>AOPP 0.670<br>CRP 0.546	18%
Renal function related factor	Creatinine 0.885<br>Urea 0.812	17%
Metabolic related factor	Glucose 0.803<br>TG 0.788	16%
Oxidative stress-hepatic related factor	MDA 0.750<br>ALT 0.713	13%

The scores originated from the PCA were used as separate variables in binary logistic regression analysis for severe psoriasis prediction, graded by PASI and DLQI scores. For the both psoriasis severity scores the cut-off value is 10, i.e. values above 10 means severe psoriasis. This cut-off value we have used for PASI score (70% of patients had PASI >10), but for the DLQI score only 3 patients had a value below 10, so we decided to use 20 as a cut-off point for extremely severe psoriasis. The P value for statistical significance was set here of 0.100.

Univariate logistic regression analysis revealed metabolic related factor as the significant PASI score (>10) predictor, while oxidative stress-hepatic related factor was significant predictor of DLQI score (>20), (Table 5).

**Table 5:** Univariate binary logistic regression analysis of PCA extracted factors for severe psoriasis prediction according to PASI and DLQI scores. Abbreviations: B – unstandardized regression weight; SE – variation of unstandardized regression weight; OR – odds ratio; CI – confidence interval

PCA<br>factors	Predictors of PASI score >10				Predictors of DLQI score > 20			
	B (SE)	Wald<br>coefficient	OR<br>(95% CI)	P	B (SE)	Wald<br>coefficient	OR<br>(95% CI)	P
Oxidative stress-<br>inflammation<br>related factor	-0.174<br>(0.403)	0.187	0.840<br>(0.382–1.849)	0.665	-0.018<br>(0.355)	0.003	0.982<br>(0.489–1.969)	0.959
Renal function<br>related factor	-0.544<br>(0.383)	2.013	0.581<br>(0.274–1.231)	0.156	-0.341<br>(0.358)	0.910	0.711<br>(0.353–1.433)	0.340
Metabolic<br>related factor	1.285<br>(0.682)	3.551	3.616<br>(0.950–13.767)	0.060	0.210<br>(0.382)	0.302	1.234<br>(0.583–2.610)	0.583
Oxidative stress-<br>hepaticrelated<br>factor	0.036<br>(0.385)	0.009	1.036<br>(0.487–2.204)	0.926	0.906<br>(0.513)	3.124	2.475<br>(0.906–6.760	0.077

The same PCA produced factors (i.e., its scores) was included in multivariate binary logistic regression analysis with backward selection mode to show a selection of factors combination which possibly show the best predictive capability towards psoriasis severity (Table 6). As the best model for PASI score >10 prediction this analysis selected a combination of renal function related factor and metabolic related factor, while oxidative stress-hepatic related factor was selected as the best predictor for DLQI score >20. All selected factors had P values below 0.100 (at the marginal level of significance), while metabolic related factor was the best model for PASI score prediction (P=0.031), which depicted this PCA factor as the most important for psoriasis severity estimation (Table 6).

**Table 6:** Multivariate binary logistic regression analysis with backward of PCA extracted factors for severe psoriasis prediction according to PASI and DLQI scores. Abbreviations: B – unstandardized regression weight; SE – variation of unstandardized regression weight; OR – odds ratio; CI – confidence interval

Predictors of PASI score >10	B (SE)	Wald coefficient	OR (95% CI)	P
Renal function related factor	-0.903 (0.478)	3.567	0.405 (0.159–1.035)	0.059
Metabolic related factor	1.794 (0.831)	4.662	6.014 (1.180–30.655)	0.031
Predictors of DLQI score > 20	B (SE)	Wald coefficient	OR (95% CI)	P
Oxidative stress-hepatic<br>related factor	0.906 (0.513)	3.124	2.475 (0.906–6.760)	0.077

## Discussion

The current study is among rare researches that examined oxidative stress, inflammation and metabolic markers in patients with psoriasis using PCA as a comprehensive statistical approach in an attempt to enlighten the knowledge gap between complex pathophysiological mechanisms underlying psoriasis and related risk factors. Herein, we have investigated several redox homeostasis biomarkers (i.e., MDA, AOPP, CAT), inflamation (i.e. CRP) and metabolic parameters (i.e., fasting glucose, lipid parameters, liver enzymes and renal function markers) in relation to psoriasis severity.

Since there is no an »ideal« redox status biomarker that can best reflect the level of oxidative stress in chronic diseases [21] [26], a large number of them were investigated in patients with psoriasis and shown contradictory results [7] [8].

Although we did not find the difference in MDA levels between psoriasis patients and healthy subjects, a significantly higher AOPP levels and antioxidant enzyme CAT activity in psoriasis patients compared to healthy counterparts were shown. Our findings are in line with Yazici et al. [17] who were the first that reported increased levels of AOPP (i.e. biomarker of proteins oxidative damage) in psoriasis. The casecontrol study of Shakoei et al. [16] with the same sample size as our study has shown similar results, but opposite to Skoie et al. [21] who did not record the difference in AOPP levels in patients with psoriasis vs. healthy controls.

AOPP reflects the overall status of proteins in the cells and are mainly obtained from oxidation- modified albumin. Namely, AOPP levels are increased in the pro-oxidant state being formed by reactions between proteins in plasma and chlorinated oxidants [27].

We did not observe the difference in serum MDA levels between examined groups, similarly to some studies [12] [13], but contrary to some other that recorded higher serum MDA levels in patients with psoriasis as compared with healthy subjects [18] [21].

Higher CAT activity in the present study might be explained by the compensatory mechanism ofantioxidants to overcome the increased production of ROS [25]. Our results are similar with Kirmit et al. [15] who found significantly higher CAT activity in patients with psoriasis than controls, but opposite to some others [19] [20] who found lower CAT in patients with psoriasis.

The conversion of H_2_O_2_ into water and oxygen is the mechanism by which CAT neutralizes this highly reactive biomolecule. Similarly to other ROS, H_2_O_2_ damages lipids, proteins and DNA [25]. The compensatory higher CAT activity in the present study could in part explain the insignificant difference in MDA between examined groups. MDA is the by-product of peroxidation of lipids (i.e., poly-unsaturated fatty acids that were damaged by ROS) formed during metabolic processes [28] [29]. The duration of the disease and the disease severity may be one of the explanations of such differences in oxidative stress biomarkers between studies. If the oxidative stress persists longer the antioxidant defense enzymes become depleted, whereas pro-oxidants are being increased. The existence of comorbidities is also a significant source of free radicals and increased oxidative stress and additional burden to pro-oxidant milieu [26] [29]. On the other hand, some medications and food consumption containing antioxidants might increase the activity and expression of antioxidative defense system [3] [21] [26].

In the current study the PCA extracted 4 factors consisted of metabolic and redox status parameters in a group of psoriasis patients as following: oxidative stress-inflammation related factor (i.e., HDL-c, CAT, AOPP and CRP), renal function related factor (i.e., creatinine and urea), metabolic related factor (i.e., glucose and TG) and oxidative stress-hepatic related factor (i.e., MDA, ALT). Indeed, previous data in literature have shown the evident interconnection between psoriasis and related comorbidities, such as dyslipidemia, insulin resistance, NAFLD, CKD [1] [9] [10] [11].

Regarding the association between oxidative stress biomarkers and severity of psoriasis there are contradictory results in the literature [12] [14] [16] [21]. We did not find any correlation between single oxidative stress biomarker and PASI or DLQI score, respectively in the current study. Similarly, some other studies [12] [14] [21] found no significant correlations between MDA and PASI, whereas the other [16] showed negative association between AOPP and PASI.

In the current study we used the scores originated from the PCA as separate variables in logistic regression analysis for severe psoriasis prediction, graded by PASI and DLQI scores. Accordingly, we have shown that a combination of metabolic related factor (i.e., glucose and TG) and renal function related (i.e., creatinine and urea) factor was shown to be the best model for PASI score >10 prediction, while oxidative stress-hepatic related factor (i.e., MDA and ALT) was selected as the best predictor for DLQI score >20.

The obtained results might be in part explained by the fact that psoriasis is a systemic inflammatory disease, with increased cytokines production that favor the alteration of biomolecules involved in the metabolism of lipids [1]. Not only that these biomolecules’ changes further affect keratinocytes in psoriasis, but they also influence the inflammation and immune response. Proinflammatory cytokines (e.g., TNF-a and IL-12/23) are shown to be related to renal inflammation and hemodynamics alterations, thus favoring sodium retention and hypertension. In addition, psoriasis is often accompanied with comorbidities such as obesity, insulin resistance, diabetes and NAFLD due to an increased levels of such proinflammatory cytokines (e.g., TNFa, IL-6 and IL-17) [1] [9] [10].

There are several strengths of the present study. Firstly, we have included age, gender and BMI matched case–control design in an attempt to eliminate potential confounding factors given the fact that age, gender and obesity influence oxidative stress and metabolic markers [26] [28]. Secondly, we have applied PCA as a comprehensive statistical approach [26] [30] to find the best cluster of biomarkers that reflect the disease severity since multimarker approach can better reflect the stratification of cardiometabolic risk [29] [31]. However, a crosssectional design of the current study does not enable us to confirm the causality between psoriasis severity and oxidative stress. Also, similarly with some other recently published articles [16] [17] [18] [19] [20], a relatively small sample size is another limititation of the study. Therefore, longitudinal studies with larger sample size are needed to further validate our results.

## Conclusions

Higher level of AOPP and CAT were shown in patients with psoriasis as compared with healthy subjects. Therefore, although several studies described oxidative stress in psoriasis and recommended the use of supportive antioxidant treatment at this stage, antioxidant treatment may also be considered in psoriasis patients since oxidative stress persists at a significant level after treatment of psoriasis as we have described in our study. Multimarker approach obtained by PCA showed that metabolic related factor (i.e., glucose and TG) and renal function related factor (i.e., creatinine and urea) were significant predictors of disease severity, i.e. PASI score (>10). Additionally, oxidative stress-hepatic related factor (i.e., MDA and ALT) was a significant predictor of DLQI score (>20). Hence, multimarker approach of these factors was better predictor of psoriasis severity than each single examined biomarker. Future studies with longitudinal design are necessary to confirm the results of the present study and to consider the treatment with antioxidative therapy in prevention and diagnostics of psoriasis.

## Dodatak

### Conflict of interest statement

All the authors declare that they have no conflict of interest in this work.
